# Simultaneously Toughening and Strengthening Soy Protein Isolate-Based Composites via Carboxymethylated Chitosan and Halloysite Nanotube Hybridization

**DOI:** 10.3390/ma10060653

**Published:** 2017-06-14

**Authors:** Xiaorong Liu, Haijiao Kang, Zhong Wang, Wei Zhang, Jianzhang Li, Shifeng Zhang

**Affiliations:** MOE Key Laboratory of Wood Material Science and Utilization, Beijing Key Laboratory of Wood Science and Engineering, College of Materials Science and Technology, Beijing Forestry University, Beijing 100083, China; happyrong1993@bjfu.edu.cn (X.L.); khj0339@bjfu.edu.cn (H.K.); wangzhong@bjfu.edu.cn (Z.W.); zhangweishe@126.com (W.Z.)

**Keywords:** soy protein isolate, halloysite nanotubes, carboxymethylated chitosan, 1,2,3-propanetriol-diglycidyl-ether, cross-linking interaction

## Abstract

Chemical cross-linking modification can significantly enhance the tensile strength (TS) of soy protein isolate (SPI)-based composites, but usually at the cost of a reduction in the elongation at break (EB). In this study, eco-friendly and high-potential hybrid SPI-based nanocomposites with improved TS were fabricated without compromising the reduction of EB. The hybrid of carboxymethylated chitosan (CMCS) and halloysite nanotubes (HNTs) as the enhancement center was added to the SPI and 1,2,3-propanetriol-diglycidyl-ether (PTGE) solution. The chemical structure, crystallinity, micromorphology, and opacity properties of the obtained SPI/PTGE/HNTs/CMCS film was analyzed by the attenuated total reflectance-Fourier transform infrared (ATR-FTIR) spectroscopy, X-ray photoelectron spectroscopy (XPS), X-ray diffraction (XRD), scanning electron microscopy (SEM), atomic force microscopy (AFM), and UV-Vis spectroscopy. The results indicated that HNTs were uniformly dispersed in the SPI matrix without crystal structure damages. Compared to the SPI/PTGE film, the TS and EB of the SPI/PTGE/HNTs/CMCS film were increased by 57.14% and 27.34%, reaching 8.47 MPa and 132.12%, respectively. The synergy of HNTs and CMCS via electrostatic interactions also improved the water resistance of the SPI/PTGE/HNTs/CMCS film. These films may have considerable potential in the field of sustainable and environmentally friendly packaging.

## 1. Introduction

In recent years, conventional petroleum-based polymers have grown increasingly unpopular amidst prevalent environmental concerns [1]. Biopolymers, such as polysaccharides, proteins, and lipids, have attracted considerable interest for their biodegradations in terms of relieving the overdependence on petroleum resources [2,3,4]. Among all biopolymers, soy protein isolate (SPI) has shown several notable advantages such as renewability, biocompatibility, biodegradability, and film-forming capacity [5]. However, the inferior mechanical properties and high moisture sensitivity of existing SPI-based films have limited their practical application [6]. The films can be modified, such as by physical treatment, chemical cross-linking, or block copolymerization, to enhance their applicability, but there is no perfect solution [7,8]. To date, chemical cross-linking has been proven to be the most effective and facile approach to enhancing the performance of SPI films [9,10].

In terms of potential applications as green, environmentally friendly composites, biocompatibility and high mechanical strength are critical factors for a given packaging film. Several researchers have explored 1,2,3-propanetriol-diglycidyl-ether (PTGE), a novel and biocompatible cross-linking agent, for this purpose [11,12]. The tensile strength (TS) of film crosslinked by PTGE was increased by 197% in one of the previous studies, but the elongation at break (EB) was reduced by 67% due to the cross-linkage effect [11]. Therefore, research on SPI-based films, to this effect, is focused on increasing the TS without compromising the reduction of EB.

Recently, a great breakthrough was made in the bio-nanocomposites field, which was the blended biopolymers with inorganic/organic nano-particles [13]. Incorporating fillers as reinforcements in the composites, such as montmorillonite, nano-SiO_2_, nano-TiO_2_, starch nanocrystal, carbon nanotubes, and rectorite nanoplatelets were the methods commonly used [14,15,16,17]. Unfortunately, most of the nanoparticles mentioned above showed bad compatibility with SPI, and need further modification.

Halloysite nanotubes (HNTs), firstly reported by Berthier in 1826, are natural aluminosilicates (Al_2_Si_2_O_5_(OH)_4_·*n*H_2_O) with nano-tubular structures [18]. Compared to other nanosized materials, naturally occurring HNTs are easily obtained and are much cheaper than other nanoparticles such as carbon nanotubes (CNTs) or boron nitride nanotubes [19]. HNTs are composed of siloxane (Si-O-Si) groups on the external surface and gibbsite octahedral sheet (Al-OH) groups on the inner surface [20]. The length of two-layered HNTs is typically 0.2–2 mm, with an external outer diameter of 40–70 nm and an inner diameter of 10–30 nm; these are especially promising candidates as reinforcing fillers due to their different chemistry at the inner and outer surfaces of the nanotube [21,22]. This difference allows for the selective modification of the material based on electrostatic interactions or specific chemical reactions [23,24]. Due to their unique tubular structure, high surface area, large aspect ratio, good biocompatibility, and low manufacturing cost, HNTs have recently received much attention in many applications [25,26]. 

Previous studies have shown that poor dispersion and lack of interfacial adhesion to the polymer matrix are the most problematic aspects of HNTs in terms of practical applications [27]. Carboxymethylated chitosan (CMCS), a water-soluble chitosan derivative, is water soluble under acidic and alkaline environments—an important physiological difference from chitosan alone, which is only soluble in acidic solutions [28]. CMCS has been widely studied due to its ease of synthesis, ampholytic characteristics, and numerous application prospects. CMCS has many reactive functional groups (e.g., amino, carboxyl, and hydroxyl groups). The interaction between carboxyl and amino groups in the CMCS structure can favor the formation of cationic–NH_3_^+^ groups, leading to a stronger electrostatic interaction with the negatively charged materials [29,30]. Therefore, CMCS is expected to effectively improve the dispersibility of HNTs, and increase its interfacial adhesion. 

The ultimate goal in conducting this study was to prepare a green and highly effective bionanocomposite packaging material via the casting method. Films with a combination of SPI, PTGE, HNTs, and CMCS were prepared and characterized by attenuated total reflectance-Fourier transform infrared (ATR-FTIR) spectroscopy, X-ray photoelectron spectroscopy (XPS), X-ray diffraction (XRD), scanning electron microscopy (SEM), atomic force microscopy (AFM), and UV-Vis spectroscopy. The films’ mechanical properties and water resistance were also investigated.

## 2. Materials and Methods

### 2.1. Materials

SPI with a protein content of 95% was purchased from Yuwang Ecological Food Industry Co., Ltd. (Shandong, China). Industrial-grade PTGE was purchased from Chuzhou Huisheng Electronic Material Co., Ltd. (Anhui, China). HNTs were obtained from Guangzhou Runwo Material Technology Co., Ltd (Guangzhou, China). CMCS was obtained from MAYA Reagent (Zhejiang, China). Other chemical reactants of analytical grade were purchased from Beijing Chemical Reagents Co., Ltd. (Beijing, China).

### 2.2. Synthesis of the Carboxymethylated Chitosan/Halloysite Nanotubes Hybrid

Crude HNTs were sieved and dried in an oven at 80 °C for 12 h, then 1.6 g HNTs and 0.8 g CMCS were respectively dispersed in 50 mL of deionized water under constant stirring for 4 h followed by probe sonication for 30 min. The CMCS polymer solution was added to the HNTs suspensions (*w/w* = 2:1) and the mixture was magnetically stirred for 12 h at room temperature.

### 2.3. Soy Protein Isolate Film Preparation

Various SPI solutions were prepared as follows. 3.0 g SPI was first dispersed in a certain amount of distilled water under stirring, and then 1.5 g glycerol was added. The SPI solution was adjusted to 9.0 pH with NaOH solution under constant stirring for 30 min and subsequently heated to 85 °C for another 30 min. PTGE was then added to the SPI solution and stirred uniformly. 

**Figure 1:**
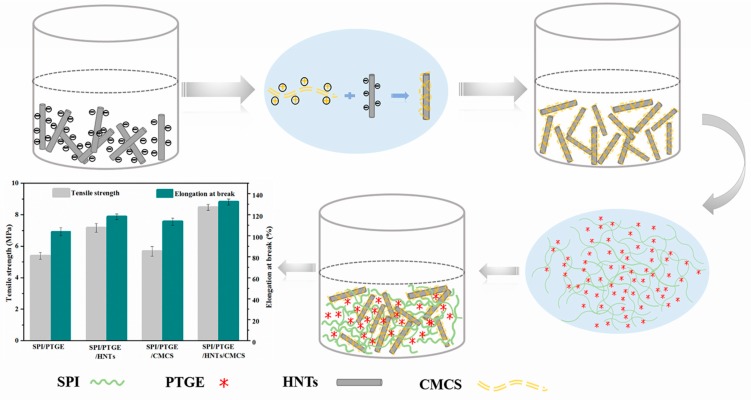
The forming process of the composite films. SPI: Soy Protein Isolate.

Certain amounts of additives (HNTs, CMCS, HNTs/CMCS) were respectively dispersed in the SPI/PTGE solution under constant stirring. The film-forming solutions (45 g) were cast onto plastic dishes, and were then dried at 45 °C for 24 h in a vacuum drying oven. After being peeled off from the plates, all films were preconditioned in the K_2_CO_3_-saturated solution in a conditioning chamber at 25 ± 2 °C and 50 ± 2% relative humidity for 48 h before testing. The composition of the modified SPI films are summarized and listed in Table 1. The forming process of the composite films is presented in Figure 1.

### 2.4. Characterization

#### 2.4.1. Attenuated Total Reflectance-Fourier Transform Infrared (ATR-FTIR) Spectroscopy

The ATR-FTIR spectra of the films was obtained by a Nicolet 6700 spectrometer (Thermo Scientific, Madison, WI, USA) equipped with an ATR accessory with a total of 32 scans at 4 cm^−1^ resolution for each sample. Measurements were recorded between 4000 and 650 cm^−1^.

#### 2.4.2. X-ray Photoelectron Spectroscopy 

XPS (Thermo Fisher Scientific Co., West Sussex, UK) was conducted with monochromatic Al Kα radiation (1486.6 eV). The X-ray beam was a 200 µm diameter beam raster over a 2 mm × 0.4 mm area on the specimens. Spectra were recorded using a pass energy of 50 eV and a resolution of 0.1 eV.

#### 2.4.3. X-ray Diffraction 

XRD was performed on a D8 advance diffractometer (Bruker AXS, Karlsruhe, Germany) equipped with a Cu Kα radiation source. The diffraction data were collected from 2θ values ranging from 5° to 60° at 2° min^−1^ at 40.0 kV and 30.0 mA.

#### 2.4.4. Scanning Electron Microscopy 

The cross-sectional morphology of different SPI films was observed with a QUANTA FEG 650 instrument (FEI, Hillsboro, OR, USA) at an acceleration voltage of 5 kV.

#### 2.4.5. Atomic Force Microscopy 

AFM (Bruker, Ettlingen, Germany) was used to observe the surface morphology of SPI-based films. The topographic (height) and phase images were recorded in tapping mode using a monolithic Si tip with a resonance frequency between 250 and 300 kHz. 

#### 2.4.6. Opacity

Opacity was measured by a UV-Vis spectrophotometer (TU-1901, Beijing Purkinje General, Beijing, China). Each film was cut into a 2.5 cm × 1 cm rectangle and opacity was determined as the area under the absorbance curve in the visible spectrum (λ = 400–800 nm).

#### 2.4.7. Water Absorption and Total Soluble Matter 

The WA test samples were dried in an oven at 103 °C for 24 h until constant weights were obtained and the dry weight (*m*_1_) was recorded. The water uptake of the films was measured by conditioning the sample in the conditioning chamber with 92% relative humidity at 25 ± 2 °C. The films were removed after 48 h and weighed on an electronic balance (*m*_2_). Five replicates were tested for each composition. WA was calculated as follows:

(1) $$\mathrm{WA}\;\left(\%\right)=\frac{\left(m_{2}\;-\;m_{1}\right)}{m_{1}}\times \;100$$

where *m*_1_ and *m*_2_ are the mass of the film before and after conditioning, respectively.

Samples were dried in the oven at 103 ± 2 °C for 24 h and reweighed (*m*_3_), and were then immersed in 30 mL distilled water with traces of sodium azide (0.02%) to prevent microbial growth and stored at 25 °C for 24 h. The samples were again dried in the oven at 103 ± 2 °C for 24 h and reweighed (*m*_4_), and then total soluble matter (TSM) was calculated as follows:

(2) $$\mathrm{TSM}\;\left(\%\right)=\frac{\left(m_{3}\;-\;m_{4}\right)}{m_{4}\;}\times \;100$$

where *m*_3_ is the dried mass and *m*_4_ is the final mass of the sample [9].

#### 2.4.8. Contact Angle Determination

Surface hydrophobicity was assessed per the sample contact angles using an OCA20 contact angle meter (Dataphysics Co., Ltd, Berlin, Germany). A film sample (20 mm × 80 mm) was placed on a movable carrier and leveled horizontally, and then a 3 μL drop of distilled water was dropped on the film surface with a microsyringe. The contact angle was measured in a conditioned room by recording the contact angle values. Each sample was tested in five replicates.

#### 2.4.9. Mechanical Properties and Coating Thickness

The mechanical properties of the SPI-based films were determined with a tensile testing machine (WDW3020, Beijing, China) according to the standard of ISO527-3:1995(E) [12]. Each sample (10 × 80 mm^2^) was tested with a speed of 20 mm min^−1^ at room temperature. The film thickness was measured with a digimatic micrometer [3].

#### 2.4.10. Statistical Analysis

The analysis of variance (ANOVA; SPSS 9.0.1, SPSS Inc, Chicago, IL, USA) was used to evaluate the significance for each sample group, which was considered as a significant difference when *p* < 0.05 [31].

## 3. Results and Discussion

### 3.1. Structural Analysis of the Soy Protein Isolate-Based Films

**Figure 2:**
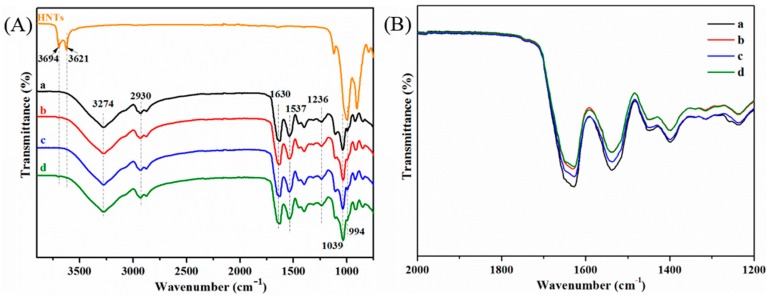
Attenuated total reflectance-Fourier transform infrared (ATR-FTIR) spectroscopy (ATR-FTIR) (**A**) of HNTs and SPI-based films: SPI and PTGE film **a**; SPI, PTGE, and HNTs film **b**; SPI, PTGE, and CMCS film **c**; SPI, PTGE, HNTs, and CMCS film **d**; magnified image (**B**) of Figure 2A from 2000 to 1200 cm^−1^.

ATR-FTIR spectra were measured to investigate the changes in functional groups in the SPI-based films. Figure 2A shows Films (a–d) shared similar spectra: The peaks at 1630, 1537, and 1236 cm^−1^ were assigned to amide I (C=O stretching), amide II N-H bending), and amide III (C-H and N-H stretching), respectively [9,10,32]. The broad absorption band at 3274 cm^−1^ was attributed to the O-H and N-H bending vibrations, and the peak at 2930 cm^−1^ was assigned to the stretching vibrations of the methylene groups. The peak situated at 1039 cm^−1^ in all spectra might be related to C-O stretching, due to the glycerol [33].

With regard to the HNTs, the peak at 3694 cm^−1^ can be attributed to the O-H stretching vibration of the external hydroxyl groups on the HNT surface; the peak at 3621 cm^−1^ was assigned to the O-H stretching vibration of the inner Al-OH groups. Other relevant peaks at 1001 and 906 cm^−1^ were associated with the stretching of Si-O and Al-OH groups, respectively [34]. After the introduction of the HNTs and CMCS (Film d) (Figure 2B), the intensities of the amide I and II bands decreased slightly due to the C=O and C-N groups in protein having undergone a physicochemical reaction with the HNTs/CMCS hybrid. Compared to Film c, there was a greater reduction in absorption peak intensity (at 1630, 1537 cm^−^^1^) in Film d, indicating that the physicochemical combination of SPI to the modifiers was more inclined to accompany the consumption of HNTs surface hydroxyl groups. In comparison to the HNTs, the characteristic peaks at 3694 and 3621 cm^−^^1^ of the HNTs nanoparticles remained in Films b and d, suggesting that the structure of HNTs was unchanged in the modified films. 

**Figure 3:**
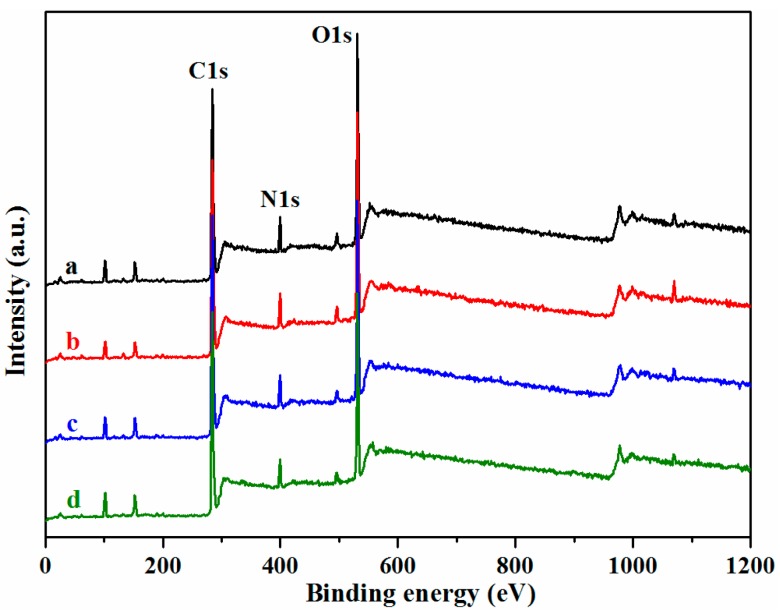
X-Ray photoelectron spectroscopy (XPS) survey spectra of the SPI and PTGE film **a**; SPI, PTGE, and HNTs film **b**; SPI, PTGE, and CMCS film **c**; SPI, PTGE, HNTs, and CMCS film **d**.

XPS analysis was also applied to investigate the surface of the films. Figure 3 shows the survey scan of the SPI-based films per the O 1s, N 1s, and C 1s peaks. Different factors can contribute to various distributions of C, O, and N on the surface. The C 1s peak was subdivided into five peaks (Figure 3) designated as C1 (C-C or C-H), C2 (C-NH-C), C3 (C-OH or C-O-C), C4 (-NH-CO-), and C5 (-COO-). The proportions of the C 1s peak areas as-obtained from the peak area computation and factor analysis are shown in Table 2.

**Figure 4:**
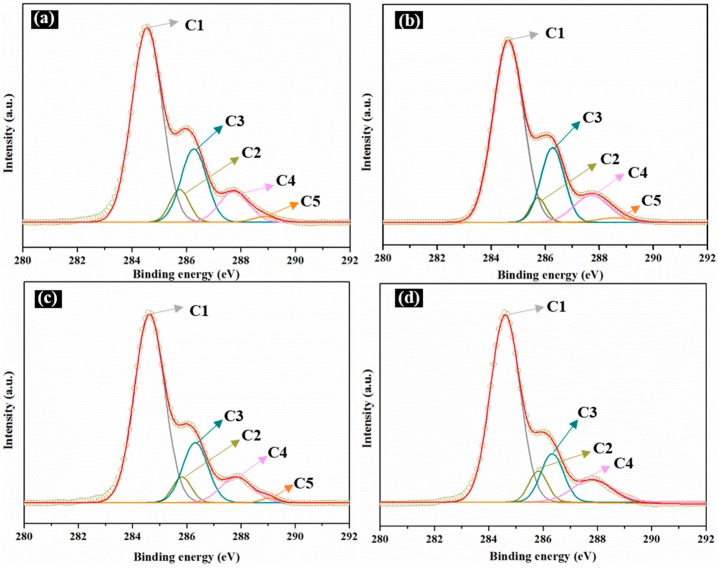
X-Ray photoelectron spectroscopy (XPS) of C 1s features of the SPI and PTGE film **a**; SPI, PTGE, and HNTs film **b**; SPI, PTGE, and CMCS film **c**; SPI, PTGE, HNTs, and CMCS film **d**.

C1 indicates the hydrophobicity of the surface; C2 and C4 reflect the cross-linking degrees, and C3 and C5 indicate the hydrophilicities [35]. As shown in Figure 4 and Table 2, the C1, C2, and C4 contents of Film d suggested greater contents than that of Film a, but lower C3 and C5 contents compared to Film a. This can be attributed to hydrogen bonding and chemical cross-linking reactions between the HNTs, CMCS, and SPI/PTGE, which further elucidated the mechanisms behind the enhanced mechanical properties and water resistance in the modified film.

**Figure 5:**
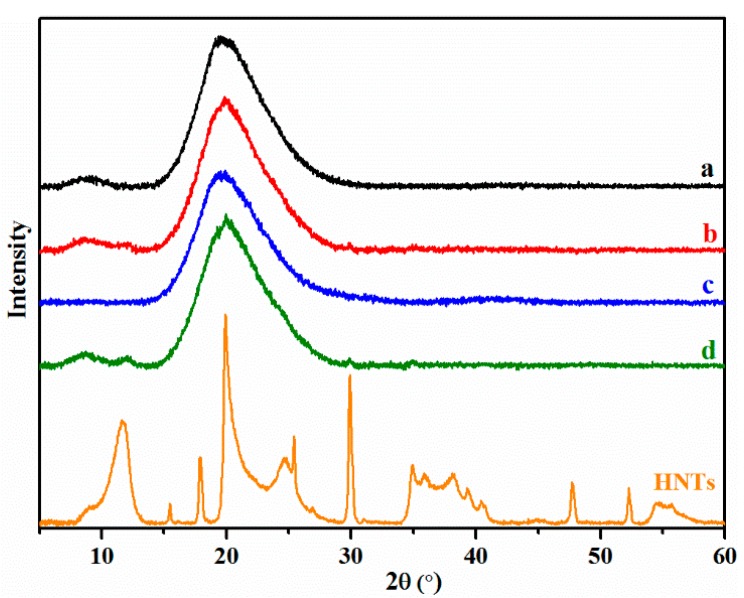
X-Ray diffraction (XRD) of the HNTs and SPI-based films: SPI and PTGE film **a**; SPI, PTGE, and HNTs film **b**; SPI, PTGE, and CMCS film **c**; SPI, PTGE, HNTs, and CMCS film **d**.

XRD is a useful technique to investigate the crystallinity of materials. Figure 5 shows peaks at 2θ = 8.6° and 19.8° corresponding to the α-helix and β-sheet structures of the SPI secondary conformation, respectively [36]. Compared to Film a, new peaks at 2θ values for Films b and d at 12°, 20°, 25°, and 30° were observed and referred to the characteristic peaks that corresponded to HNTs. The obvious diffraction peak of HNTs was visible due to the crystalline structure of typical silicate materials [37,38,39]. The basal reflection of 7.25 Å indicated that the used HNTs were dehydrated. However, the reflection peak at 2θ = 20° of Film d might be superimposed compared with Film a, implying that the crystal structures of HNTs were not significantly altered. 

### 3.2. Soy Protein Isolate Film Micromorphology

**Figure 6:**
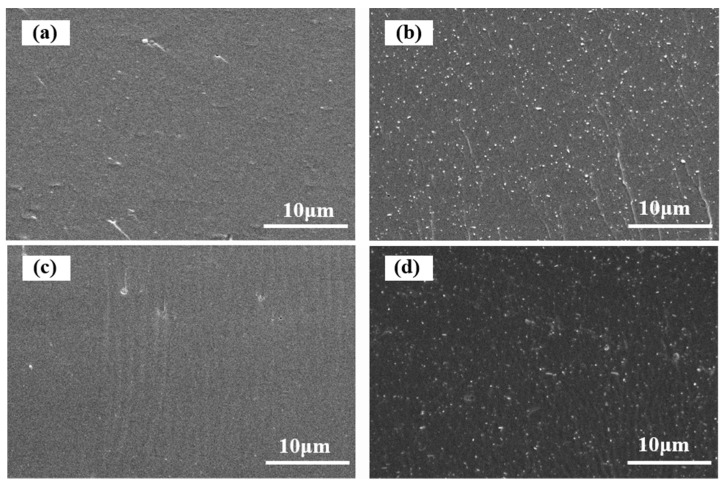
Scanning electron Microscopy (SEM) images of the SPI and PTGE film **a**; SPI, PTGE, and HNTs film **b**; SPI, PTGE, and CMCS film **c**; SPI, PTGE, HNTs, and CMCS film **d**.

The morphology of the sample films was also examined by SEM observation. Figure 6 shows the fracture surface micrographs of the SPI-based films. The uniformly distributed white dots in Figure 6b reflect favorable dispersion for the loading of HNTs into the matrix. When CMCS was introduced into Film a, the cross-section exhibited a clean, dense, and uniform surface due to high miscibility between the SPI and CMCS [40]. In comparison with Film c, the interface between HNTs and the matrix in Film d was slightly blurred, suggesting improved interfacial bonding after CMCS was introduced [41]. This observation corresponds to the improvement in the films’ mechanical properties, which is discussed in detail below.

**Figure 7:**
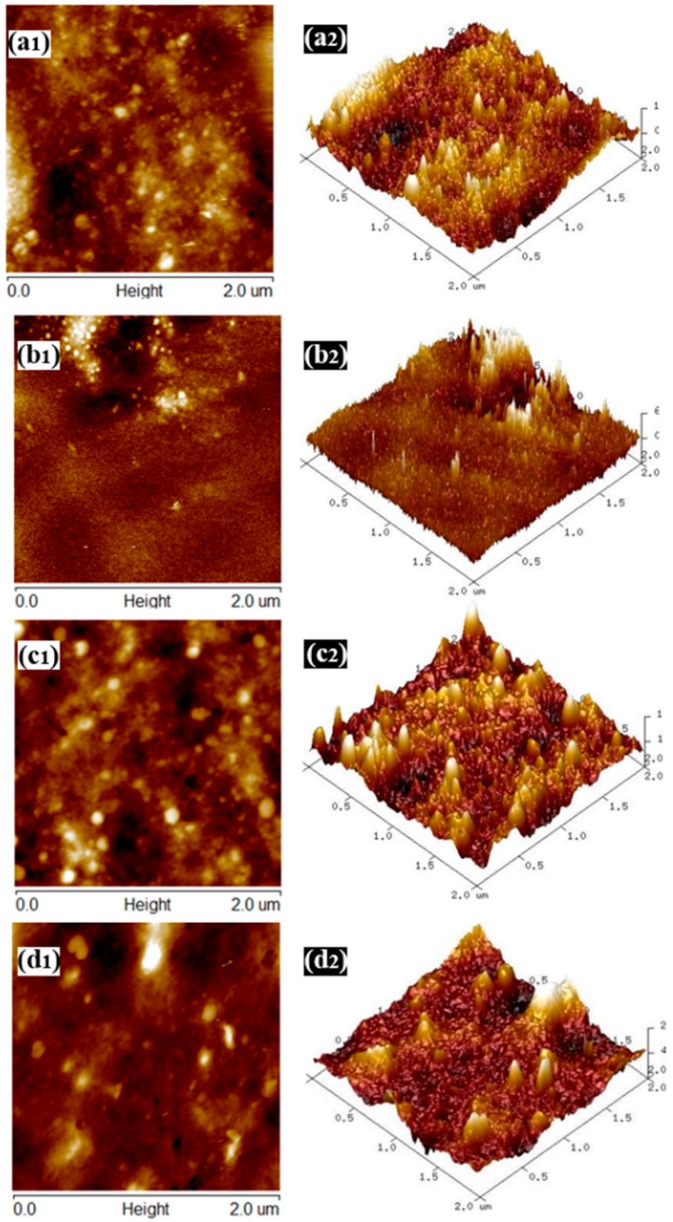
Atomic force microscopy (AFM) height images (**a1**–**d1**) and the 3D topography (**a2**–**d2**) of films **a**–**d**.

The nanoscale topography for the surface of the SPI-based films was also explored by AFM. The height images and 3D topography of the films are shown in Figure 7.

Film a exhibited a relatively rough surface due to the self-aggregation of the soy protein molecules. The root-mean-square (RMS) roughness of Films a–d (2 μm × 2 μm area calculation) were 3.09, 1.25, 3.61 and 5.14 nm, respectively, indicating that the presence of HNTs and CMCS contributed to an increase in the surface roughness of Film d due to the discontinuous filling of nanotubes. The surface needlepoints on Film a were also mostly shaved away after incorporating HNTs and CMCS (Film d), suggesting favorable interfacial interactions between the CMCS pre-dispersed HNTs and the matrix [3,39].

### 3.3. Opacity

**Figure 8:**
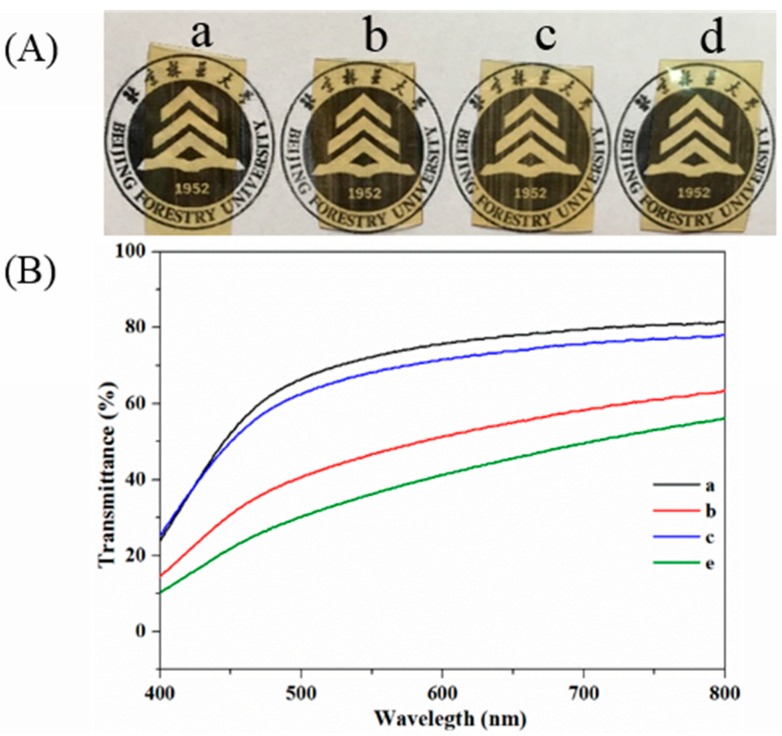
Photographs (**A**) and UV-Vis spectra (**B**) of the SPI-based films: SPI and PTGE film **a**; SPI, PTGE, and HNTs film **b**; SPI, PTGE, and CMCS film **c**; SPI, PTGE, HNTs, and CMCS film **d**.

The transparency of the SPI-based films was examined by UV-Vis spectrophotometer. As shown in Figure 8A, Films b–d retained their transparency well after the modification, indicating a homogeneous distribution of HNTs in the SPI matrix. The UV-Vis spectra of the SPI-based films are shown in Figure 8B. The transmission of Film a was 81.4% at a wavelength of 800 nm, while Film d was 56.1%, suggesting that the incorporation of HNTs enhanced visible light barrier performance, which is important for food packaging [42].

### 3.4. Mechanical Properties of the Composite Films

Chemical cross-linking modification can significantly enhance the TS of SPI-based films with sacrificing a film’s EB [10]. TS and EB are, as discussed above, critical mechanical properties for packaging materials. The TS and EB of our film samples are summarized as shown in Table 3. The SPI composite films of intermolecular disulfide bonding, hydrogen bonding, and electrostatic forces between protein chains typically leads to brittle films [11]. With the addition of glycerol, the SPI-based films obtained are flexible and they present good mechanical properties. The use of glycerol to break the intermolecular linkage that stabilizes the proteins in their primitive structures makes the protein chains mobile [12,13]. Compared to Film a, the TS of Film b increased from 5.39 to 7.16 MPa, an increase of 32.84% (*p* > 0.05). This likely resulted from the discontinuous filling of nanoscaled HNTs into the SPI matrix and partly restricting the SPI chains’ movement [43]. In the CMCS modified Film a, the TS and EB increased to 5.68 MPa and 113.42% (5.38% and 9.32%) (*p* > 0.05), respectively, marking a limited impact on the mechanical properties of the SPI/PTGE-based film [44]. The TS and EB values of Film d demonstrated the greatest increments by 57.14% and 27.34%, reaching 8.47 MPa and 132.12%, respectively, compared to the control ones. This can be attributed to the improved dispersion of HNTs and their interfacial combinations to the SPI matrix with the aid of CMCS (Film d), as well as the bulk cross-linking network among HNTs, CMCS, and SPI/PTGE.

### 3.5. Water Resistance and Surface Hydrophilicity Properties

The effect of diverse additives on the water resistance (WA and TSM) and surface hydrophilicity (WCA) of the SPI-based films is listed in Table 4. Both WA and TSM decreased substantially after HNTs or CMCS were introduced. In Film b, it was found that the addition of HNTs caused a decrease in WA and TSM likely because the HNTs increased the film’s tortuosity (*p* > 0.05), leading to a slower diffusion of water molecules through the film matrix [45,46,47]. The addition of CMCS caused a decrease in the WA and TSM of the films (Film c). The decrease in WA and TSM values from 64.10% to 60.50% and from 31.27% to 30.14% (*p* > 0.05), respectively, may be attributed to the formation of hydrogen bonds between CMCS and the SPI/PTGE matrix, resulting in a reduced number of hydroxyl groups in the film [48]. The WCA of Film d was the highest among the films, most likely because of the existence of HNTs uniformly dispersed in the matrix with the aid of CMCS. These results altogether suggested that the addition of HNTs and CMCS could improve the water resistance of SPI/PTGE-based films (Films a–d) through the improved interfacial combinations and physical/chemical cross-linking reactions.

## 4. Conclusions

A green and effective biopolymer/nano-inorganic packaging material (SPI/PTGE/HNTs/CMCS film) was prepared in this study. With the assistance of CMCS, not only HNTs were well dispersed throughout the material, but also the interface adhesion of HNTs was improved due to the hydrogen bonding and electrostatic interaction between the two fillers. As a result, the TS and EB of the SPI/PTGE/HNTs/CMCS film increased from 5.39 MPa to 8.47 MPa and 103.75% to 132.12%, respectively, compared to those of the SPI/PTGE films. The WA and TSM of the SPI/PTGE/HNTs/CMCS film also decreased from 64.10% to 61.34% and from 31.27% to 29.98%, respectively. These results altogether imply that SPI/PTGE/HNTs/CMCS nanocomposite films have notable potential as novel and eco-friendly packaging materials.

## Figures and Tables

**Table 1:** Different formulations of Soy Protein Isolate (SPI)-based films a–d.

Sample	SPI (g)	Glycerol (g)	Water (g)	PTGE (g)	HNTs (g)	CMCS (g)
a	3	1.5	57	0.3	0	0
b	3	1.5	57	0.3	0.09	0
c	3	1.5	57	0.3	0	0.09
d	3	1.5	57	0.3	0.09	0.09

Note: PTGE: 1,2,3-propanetriol-diglycidyl-ether; HNTs: Halloysite nanotubes; CMCS: Carboxymethylated chitosan.

**Table 2:** Relative amounts (%) of carbon (C 1s) on the surfaces of films a–d.

Sample	C1 (%)	C2 (%)	C3 (%)	C4 (%)	C5 (%)
a	64.42	6.67	19.88	9.66	1.37
b	63.06	4.45	20.00	10.76	1.73
c	66.43	5.59	17.70	9.46	0.82
d	68.26	7.09	13.55	11.09	0

**Table 3:** Thickness and mechanical properties of the SPI-based films a–d.

Sample	Thickness (mm) Mean (SD)	Tensile Strength (MPa) Mean (SD)	Elongation at Break (%) Mean (SD)
a	0.26 (0.013)	5.39 (0.22)	103.75 (3.82)
b	0.25 (0.011)	7.16 (0.27)	118.10 (2.64)
c	0.20 (0.015)	5.68 (0.31)	113.42 (3.38)
d	0.23 (0.009)	8.47 (0.19)	132.12 (2.75)
Increment (%) ^a^	-	57.14	27.34

^a^ Increment calculated from the mechanical properties of film d compared to film a. SD: Standard deviation.

**Table 4:** Water Absorption (WA), Total Soluble Matter (TSM), and Water Contact Angles (WCA) of the SPI-based films a–d.

Sample	Water Absorption (%) Mean (SD)	Total Soluble Matter (%) Mean (SD)	Water Contact Angles (°) Mean (SD)
a	64.10 (1.78)	31.27 (0.98)	34.83 (2.24)
b	63.02 (1.26)	29.06 (1.45)	36.23 (1.97)
c	60.50 (2.01)	30.14 (1.36)	31.83 (0.87)
d	61.34 (1.15)	29.98 (1.77)	38.96 (1.42)
Increment (%) ^a^	−4.31	−4.13	11.86

^a^ Increment calculated from the water resistance of film d compared to film a.
